# Response to the Letter to the Editor

**DOI:** 10.3109/02813432.2013.846999

**Published:** 2013-12

**Authors:** Jozé Braspenning, Renate Jansink

**Affiliations:** Scientific Institute for Quality of Healthcare, Radboud University Nijmegen Medical Centre, PO Box 9101, Nijmegen, 6500 HB, The Netherlands. Email: r.jansink@iq.umcn.nl

Adams et al. have some concerns about the interpretation of the results of our trial. In the paper “No identifiable HbA1c or lifestyle change after a comprehensive diabetes programme including motivational interviewing: A cluster randomized trial” [1] we considered several options to explain our findings which relate to the unease. First of all, we stated that the level of diabetes management in The Netherlands was perhaps too high to expect results that could be attributed to our programme. Second, we discussed that our education programme could be insufficient. We based our training programme on the study of Rubak et al. in general practices, which showed a positive effect of motivational interviewing (MI) on professional behaviour [2]. Our nurses did receive reminders and feedback, and although the participation at the follow-up meeting was low (37%), all nurses received three quarterly telephone follow-ups from the research team and they could call the research team at any time for information and questions. In the discussion section, we argued that more training on the job probably can help to produce more effective lifestyle counselling [3]. Third, we submitted the idea to organize a setting that is explicitly dedicated to MI, because separate MI sessions have been shown to be successful [4]. As a fourth point we raised the question of whether MI could be more effective for less complicated conditions than diabetes mellitus, and related to this we made the suggestion to invest in personalized lifestyle counselling as MI is probably more suitable for only some patients. So, a number of the arguments raised are discussed in the paper itself.

However, we do not get the argument on agenda setting and record keeping. The literature showed that techniques such as agenda setting, scaling questions, and assessing the importance and confidence in changing lifestyle can be used to support MI [5]. As MI had to become embedded in normal care, we advised record keeping. We expected MI to be stimulated by these aspects or at least that the MI robustness should be unaffected. Furthermore, we would like to apologize for the confusion but no lifestyle counselling techniques other than MI were introduced in our study.

Finally, Adams et al. maintain that we did not take into account objective observation to ensure nurses’ fidelity to the principles of MI. In a separate paper [6], we reported MI skills based on 340 videos of nurse consultations. We believe that this activity informs us regarding the nurses’ fidelity, because we rated actual behaviour. Few studies have reported on actual MI behaviour so extensively. Nevertheless, it was demonstrated that the nurses improved only minimally in their MI skills. As suggested above more education and a more tailored setting might improve MI skills.

In short, just like Adams et al. we were wondering why our comprehensive programme showed no effect on diabetes management. The video study indicated that the nurses hardly practised MI during the intervention period, but the other explanations could have contributed to our study outcome as well.
